# 1-Methyl­amino-3-(2,4,6-trimethyl­phen­yl)propan-2-ol

**DOI:** 10.1107/S1600536811007513

**Published:** 2011-03-05

**Authors:** Abel M. Maharramov, Ali N. Khalilov, Atash V. Gurbanov, Mirze A. Allahverdiyev, Seik Weng Ng

**Affiliations:** a Department of Organic Chemistry, Baku State University, Baku, Azerbaijan; b Department of Chemistry, University of Malaya, 50603 Kuala Lumpur, Malaysia

## Abstract

The methyl­amino­propyl chain in the title compound, C_13_H_21_NO, adopts an extended zigzag conformation and the N atom shows a trigonal coordination. The N atom acts as hydrogen-bond acceptor to the hy­droxy group of an adjacent mol­ecule, generating a helical chain running along the *b* axis. The amino H atom is not involved in hydrogen bonding.

## Related literature

For background to the synthesis: see: Yadigarov *et al.* (2010 ▶). For the structure of 1-(piperidin-1-yl)-3-(2,4,6-trimethyl­phen­yl)propan-2-ol, see: Maharramov *et al.* (2011 ▶).
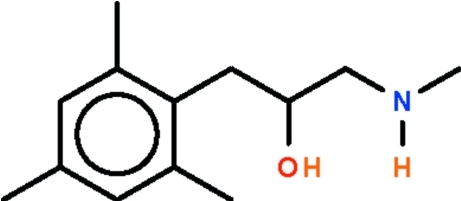


## Experimental

### 

#### Crystal data

C_13_H_21_NO
*M* *_r_* = 207.31
Monoclinic, 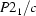

*a* = 14.408 (1) Å
*b* = 5.8150 (4) Å
*c* = 14.4503 (10) Å
β = 91.371 (1)°
*V* = 1210.34 (14) Å^3^
*Z* = 4
Mo *K*α radiation
μ = 0.07 mm^−1^
*T* = 100 K
0.30 × 0.20 × 0.20 mm

#### Data collection

Bruker APEXII diffractometer
9964 measured reflections
2775 independent reflections
2384 reflections with *I* > 2σ(*I*)
*R*_int_ = 0.019

#### Refinement

*R*[*F* ^2^ > 2σ(*F* ^2^)] = 0.038
*wR*(*F* ^2^) = 0.111
*S* = 1.04
2775 reflections
148 parameters
2 restraints
H atoms treated by a mixture of independent and constrained refinement
Δρ_max_ = 0.29 e Å^−3^
Δρ_min_ = −0.15 e Å^−3^

### 

Data collection: *APEX2* (Bruker, 2005 ▶); cell refinement: *SAINT* (Bruker, 2005 ▶); data reduction: *SAINT*; program(s) used to solve structure: *SHELXS97* (Sheldrick, 2008 ▶); program(s) used to refine structure: *SHELXL97* (Sheldrick, 2008 ▶); molecular graphics: *X-SEED* (Barbour, 2001 ▶); software used to prepare material for publication: *publCIF* (Westrip, 2010 ▶).

## Supplementary Material

Crystal structure: contains datablocks global, I. DOI: 10.1107/S1600536811007513/bt5484sup1.cif

Structure factors: contains datablocks I. DOI: 10.1107/S1600536811007513/bt5484Isup2.hkl

Additional supplementary materials:  crystallographic information; 3D view; checkCIF report

## Figures and Tables

**Table 1:** Hydrogen-bond geometry (Å, °)

*D*—H⋯*A*	*D*—H	H⋯*A*	*D*⋯*A*	*D*—H⋯*A*
O1—H1⋯N1^i^	0.87 (1)	1.93 (1)	2.789 (1)	173 (2)

Symmetry code: (i) 
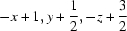
.

**Fig. 1.**
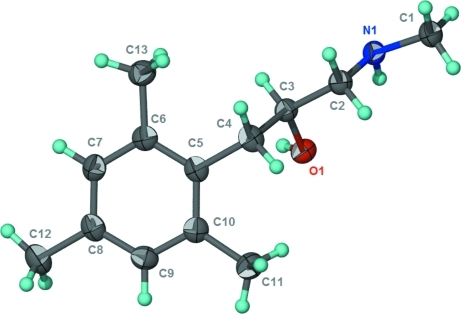
Anisotropic displacement ellipsoid plot (Barbour, 2001) of C13H21NO at the 70% probability level; hydrogen atoms are drawn as spheres of arbitrary radius.

## supplementary crystallographic information

### Comment

A recent study reported the reaction of 1-chloro-3-(2,4,6-trimethylphenyl)propan-2-one and primary amines. The chlorine atom in the α-chloro ketone is not replaced directly by the amino RNH– group; the intermediate product undergoes a Favorskii rearrangement that furnishes a compound having two methylene groups between the aromatic system and the amido unit (Yadigarov *et al.*, 2010). A recent study used cyclic secondary amine as the amino reactant in the synthesis of a compound having a formulation similar to that of tolperisone (a piperidine derivative that is commercially used as a muscle relaxant) (Maharramov *et al.* 2011). The present study uses the methylamine to yield the C_13_H_21_NO molecule (Scheme I). The methylaminopropyl chain of C_13_H_27_NO adopts an extended zigzag conformation and the N atom shows trigonal coordination (Fig. 1). The N atom acts as hydrogen-bond acceptor to the hydroxy group of an adjacent molecule to generate a helical chain running along the *b*-axis of the monoclinic unit cell.

### Experimental

1-Chloro-3-(2,4,6-trimethylphenyl)propan-2-one (1 mol) and piperidine (1 mmol) were stirred in water for 18 h at 53 K. The water was decanted and the oil was distilled in vacuum. The distillate was a liquid; the liquid crystallized after 6 months; yield 70%.

### Refinement

Carbon-bound H-atoms were placed in calculated positions [C–H 0.95 to 1.00 Å; *U*(H) 1.2 to 1.5*U*(C)] and were included in the refinement in the riding model approximation.

The hydroxy and amino H-atoms were located in a difference Fourier map, and were refined with distance restraints of O–H 0.84±0.01 and N–H 0.88±0.01 Å.

### Crystal data

**Table:**

C_13_H_21_NO	*F*(000) = 456
*M**_r_* = 207.31	*D*_x_ = 1.138 Mg m^−^^3^
Monoclinic, *P*2_1_/*c*	Mo *K*α radiation, λ = 0.71073 Å
Hall symbol: -P 2ybc	Cell parameters from 4648 reflections
*a* = 14.408 (1) Å	θ = 2.8–28.3°
*b* = 5.8150 (4) Å	µ = 0.07 mm^−^^1^
*c* = 14.4503 (10) Å	*T* = 100 K
β = 91.371 (1)°	Block, colorless
*V* = 1210.34 (14) Å^3^	0.30 × 0.20 × 0.20 mm
*Z* = 4	

### Data collection

**Table:**

Bruker APEXII diffractometer	2384 reflections with *I* > 2σ(*I*)
Radiation source: fine-focus sealed tube	*R*_int_ = 0.019
graphite	θ_max_ = 27.5°, θ_min_ = 2.8°
φ and ω scans	*h* = −18→18
9964 measured reflections	*k* = −7→7
2775 independent reflections	*l* = −18→18

### Refinement

**Table:**

Refinement on *F*^2^	Primary atom site location: structure-invariant direct methods
Least-squares matrix: full	Secondary atom site location: difference Fourier map
*R*[*F*^2^ > 2σ(*F*^2^)] = 0.038	Hydrogen site location: inferred from neighbouring sites
*wR*(*F*^2^) = 0.111	H atoms treated by a mixture of independent and constrained refinement
*S* = 1.04	*w* = 1/[σ^2^(*F*_o_^2^) + (0.0562*P*)^2^ + 0.3427*P*] where *P* = (*F*_o_^2^ + 2*F*_c_^2^)/3
2775 reflections	(Δ/σ)_max_ = 0.001
148 parameters	Δρ_max_ = 0.29 e Å^−^^3^
2 restraints	Δρ_min_ = −0.15 e Å^−^^3^

### Fractional atomic coordinates and isotropic or equivalent isotropic displacement parameters (Å^2^)

**Table:**

	*x*	*y*	*z*	*U*_iso_*/*U*_eq_	
O1	0.40834 (5)	0.62853 (13)	0.68021 (5)	0.02485 (19)	
H1	0.4176 (12)	0.690 (3)	0.7345 (8)	0.054 (5)*	
N1	0.54856 (6)	0.29883 (16)	0.64351 (6)	0.0236 (2)	
H2	0.5558 (9)	0.4495 (15)	0.6437 (9)	0.032 (4)*	
C1	0.60577 (8)	0.1994 (2)	0.57080 (8)	0.0292 (3)	
H1A	0.6715	0.2278	0.5856	0.044*	
H1B	0.5893	0.2709	0.5112	0.044*	
H1C	0.5947	0.0334	0.5671	0.044*	
C2	0.44967 (7)	0.25622 (18)	0.62563 (7)	0.0240 (2)	
H2A	0.4369	0.0898	0.6320	0.029*	
H2B	0.4332	0.3018	0.5613	0.029*	
C3	0.39001 (7)	0.38978 (18)	0.69225 (7)	0.0222 (2)	
H3	0.4073	0.3446	0.7572	0.027*	
C4	0.28756 (7)	0.3311 (2)	0.67318 (8)	0.0265 (2)	
H4A	0.2725	0.3660	0.6075	0.032*	
H4B	0.2792	0.1636	0.6820	0.032*	
C5	0.21865 (7)	0.45598 (19)	0.73277 (7)	0.0233 (2)	
C6	0.19897 (7)	0.37481 (19)	0.82164 (7)	0.0246 (2)	
C7	0.13219 (7)	0.4868 (2)	0.87371 (7)	0.0260 (2)	
H7	0.1190	0.4302	0.9337	0.031*	
C8	0.08455 (7)	0.6778 (2)	0.84056 (8)	0.0261 (2)	
C9	0.10519 (8)	0.75788 (19)	0.75269 (8)	0.0266 (2)	
H9	0.0734	0.8891	0.7289	0.032*	
C10	0.17130 (7)	0.65081 (19)	0.69854 (7)	0.0246 (2)	
C11	0.18859 (9)	0.7448 (2)	0.60308 (8)	0.0316 (3)	
H11A	0.1554	0.8907	0.5949	0.047*	
H11B	0.1664	0.6344	0.5564	0.047*	
H11C	0.2553	0.7704	0.5960	0.047*	
C12	0.01217 (8)	0.7968 (2)	0.89726 (9)	0.0340 (3)	
H12A	0.0111	0.7277	0.9591	0.051*	
H12B	−0.0489	0.7795	0.8668	0.051*	
H12C	0.0274	0.9606	0.9028	0.051*	
C13	0.24826 (8)	0.1699 (2)	0.86386 (8)	0.0303 (3)	
H13A	0.2144	0.1166	0.9179	0.045*	
H13B	0.3115	0.2139	0.8831	0.045*	
H13C	0.2509	0.0459	0.8180	0.045*	

### Atomic displacement parameters (Å^2^)

**Table:**

	*U*^11^	*U*^22^	*U*^33^	*U*^12^	*U*^13^	*U*^23^
O1	0.0301 (4)	0.0223 (4)	0.0221 (4)	−0.0012 (3)	−0.0007 (3)	−0.0008 (3)
N1	0.0252 (5)	0.0231 (5)	0.0225 (4)	0.0006 (4)	0.0033 (3)	−0.0003 (3)
C1	0.0308 (6)	0.0302 (6)	0.0268 (5)	0.0043 (5)	0.0060 (4)	−0.0012 (4)
C2	0.0263 (5)	0.0241 (5)	0.0217 (5)	0.0004 (4)	0.0004 (4)	−0.0024 (4)
C3	0.0242 (5)	0.0218 (5)	0.0206 (5)	−0.0002 (4)	0.0000 (4)	−0.0008 (4)
C4	0.0254 (5)	0.0274 (5)	0.0267 (5)	−0.0013 (4)	−0.0003 (4)	−0.0061 (4)
C5	0.0199 (5)	0.0254 (5)	0.0245 (5)	−0.0033 (4)	−0.0018 (4)	−0.0045 (4)
C6	0.0211 (5)	0.0252 (5)	0.0273 (5)	−0.0039 (4)	−0.0028 (4)	−0.0009 (4)
C7	0.0222 (5)	0.0323 (6)	0.0235 (5)	−0.0045 (4)	−0.0001 (4)	0.0000 (4)
C8	0.0197 (5)	0.0316 (6)	0.0270 (5)	−0.0020 (4)	−0.0011 (4)	−0.0060 (4)
C9	0.0237 (5)	0.0278 (5)	0.0282 (5)	0.0013 (4)	−0.0045 (4)	−0.0011 (4)
C10	0.0236 (5)	0.0280 (5)	0.0221 (5)	−0.0029 (4)	−0.0033 (4)	−0.0027 (4)
C11	0.0339 (6)	0.0369 (6)	0.0240 (5)	0.0013 (5)	−0.0023 (4)	0.0015 (5)
C12	0.0267 (6)	0.0412 (7)	0.0342 (6)	0.0045 (5)	0.0030 (5)	−0.0061 (5)
C13	0.0285 (6)	0.0284 (6)	0.0339 (6)	−0.0016 (4)	−0.0003 (5)	0.0047 (5)

### Geometric parameters (Å, °)

**Table:**

O1—C3	1.4247 (13)	C6—C7	1.3966 (15)
O1—H1	0.870 (9)	C6—C13	1.5087 (16)
N1—C2	1.4632 (14)	C7—C8	1.3854 (16)
N1—C1	1.4693 (14)	C7—H7	0.9500
N1—H2	0.882 (9)	C8—C9	1.3912 (16)
C1—H1A	0.9800	C8—C12	1.5094 (15)
C1—H1B	0.9800	C9—C10	1.3939 (15)
C1—H1C	0.9800	C9—H9	0.9500
C2—C3	1.5193 (14)	C10—C11	1.5100 (15)
C2—H2A	0.9900	C11—H11A	0.9800
C2—H2B	0.9900	C11—H11B	0.9800
C3—C4	1.5334 (14)	C11—H11C	0.9800
C3—H3	1.0000	C12—H12A	0.9800
C4—C5	1.5149 (15)	C12—H12B	0.9800
C4—H4A	0.9900	C12—H12C	0.9800
C4—H4B	0.9900	C13—H13A	0.9800
C5—C6	1.4036 (15)	C13—H13B	0.9800
C5—C10	1.4062 (15)	C13—H13C	0.9800
			
C3—O1—H1	108.4 (11)	C7—C6—C13	118.31 (10)
C2—N1—C1	111.57 (9)	C5—C6—C13	122.15 (10)
C2—N1—H2	106.4 (9)	C8—C7—C6	121.98 (10)
C1—N1—H2	109.0 (9)	C8—C7—H7	119.0
N1—C1—H1A	109.5	C6—C7—H7	119.0
N1—C1—H1B	109.5	C7—C8—C9	117.96 (10)
H1A—C1—H1B	109.5	C7—C8—C12	121.53 (10)
N1—C1—H1C	109.5	C9—C8—C12	120.51 (11)
H1A—C1—H1C	109.5	C8—C9—C10	121.79 (10)
H1B—C1—H1C	109.5	C8—C9—H9	119.1
N1—C2—C3	111.40 (8)	C10—C9—H9	119.1
N1—C2—H2A	109.3	C9—C10—C5	119.65 (10)
C3—C2—H2A	109.3	C9—C10—C11	118.75 (10)
N1—C2—H2B	109.3	C5—C10—C11	121.59 (10)
C3—C2—H2B	109.3	C10—C11—H11A	109.5
H2A—C2—H2B	108.0	C10—C11—H11B	109.5
O1—C3—C2	108.14 (8)	H11A—C11—H11B	109.5
O1—C3—C4	112.04 (9)	C10—C11—H11C	109.5
C2—C3—C4	109.23 (8)	H11A—C11—H11C	109.5
O1—C3—H3	109.1	H11B—C11—H11C	109.5
C2—C3—H3	109.1	C8—C12—H12A	109.5
C4—C3—H3	109.1	C8—C12—H12B	109.5
C5—C4—C3	115.63 (9)	H12A—C12—H12B	109.5
C5—C4—H4A	108.4	C8—C12—H12C	109.5
C3—C4—H4A	108.4	H12A—C12—H12C	109.5
C5—C4—H4B	108.4	H12B—C12—H12C	109.5
C3—C4—H4B	108.4	C6—C13—H13A	109.5
H4A—C4—H4B	107.4	C6—C13—H13B	109.5
C6—C5—C10	119.07 (10)	H13A—C13—H13B	109.5
C6—C5—C4	120.56 (10)	C6—C13—H13C	109.5
C10—C5—C4	120.34 (10)	H13A—C13—H13C	109.5
C7—C6—C5	119.54 (10)	H13B—C13—H13C	109.5
			
C1—N1—C2—C3	−171.17 (9)	C13—C6—C7—C8	179.21 (10)
N1—C2—C3—O1	60.20 (11)	C6—C7—C8—C9	−0.18 (16)
N1—C2—C3—C4	−177.63 (9)	C6—C7—C8—C12	179.70 (10)
O1—C3—C4—C5	−58.72 (12)	C7—C8—C9—C10	0.23 (16)
C2—C3—C4—C5	−178.53 (9)	C12—C8—C9—C10	−179.64 (10)
C3—C4—C5—C6	−82.99 (12)	C8—C9—C10—C5	0.23 (16)
C3—C4—C5—C10	99.05 (12)	C8—C9—C10—C11	178.81 (10)
C10—C5—C6—C7	0.81 (15)	C6—C5—C10—C9	−0.75 (15)
C4—C5—C6—C7	−177.18 (9)	C4—C5—C10—C9	177.24 (9)
C10—C5—C6—C13	−178.73 (10)	C6—C5—C10—C11	−179.29 (10)
C4—C5—C6—C13	3.28 (15)	C4—C5—C10—C11	−1.30 (15)
C5—C6—C7—C8	−0.35 (16)		

### Hydrogen-bond geometry (Å, °)

**Table:**

*D*—H···*A*	*D*—H	H···*A*	*D*···*A*	*D*—H···*A*
O1—H1···N1^i^	0.87 (1)	1.93 (1)	2.789 (1)	173 (2)

Symmetry codes: (i) −*x*+1, *y*+1/2, −*z*+3/2.
